# Isolated anti-Ro52 identifies a severe subset of Sjögren’s syndrome patients

**DOI:** 10.3389/fimmu.2023.1115548

**Published:** 2023-03-16

**Authors:** Adrian Y. S. Lee, Trishni Putty, Ming-Wei Lin, Sanjay Swaminathan, Dan Suan, Tim Chataway, Rogier M. Thurlings, Tom P. Gordon, Jing Jing Wang, Joanne H. Reed

**Affiliations:** ^1^ Centre for Immunology and Allergy Research, Westmead Institute for Medical Research, The University of Sydney, Westmead, NSW, Australia; ^2^ Department of Clinical Immunology and Allergy, Westmead Hospital and Institute of Clinical Pathology & Medical Research (ICPMR), Westmead, NSW, Australia; ^3^ Department of Immunology, Flinders University, Bedford Park, SA, Australia; ^4^ Department of Immunology, SA Pathology, Flinders Medical Centre, Bedford Park, SA, Australia; ^5^ Flinders Proteomic Facility, Flinders University, Bedford Park, SA, Australia; ^6^ Department of Rheumatology, Radboud University Medical Center, Nijmegen, Netherlands

**Keywords:** anti-Ro52/TRIM21, autoantibodies, cryoglobulinaemia, rheumatoid factor, Ro/La, Sjögren’s syndrome

## Abstract

**Introduction:**

Serum autoantibodies targeting the SSA/Ro proteins are a key component of the classification criteria for the diagnosis of Sjögren’s syndrome (SS). Most patients' serum reacts with both Ro60 and Ro52 proteins. Here we compare the molecular and clinical characteristics of patients diagnosed with SS with anti-Ro52 in the presence or absence of anti-Ro60/La autoantibodies.

**Methods:**

A cross-sectional study was performed. Patients in the SS biobank at Westmead Hospital (Sydney, Australia) that were positive for anti-Ro52 were included and stratified based on the absence (isolated) or presence (combined) of anti-Ro60/La, measured by line immunoassay. We examined clinical associations and the serological and molecular characteristics of anti-Ro52 using ELISA and mass spectrometry in serological groups.

**Results:**

A total of 123 SS patients were included for study. SS patients with isolated anti-Ro52 (12%) identified a severe serological subset characterised by higher disease activity, vasculitis, pulmonary involvement, rheumatoid factor (RhF) and cryoglobulinaemia. Serum antibodies reacting with Ro52 in the isolated anti-Ro52 subset displayed less isotype switching, less immunoglobulin variable region subfamily usage and a lower degree of somatic hypermutation than the combined anti-Ro52 subset.

**Conclusions:**

In our cohort of SS patients, isolated anti-Ro52 represents a severe subset of SS, and is associated with the presence of cryoglobulinaemia. We therefore provide clinical relevance to the stratification of SS patients by their sero-reactivities. It is possible that the autoantibody patterns may be immunological epiphenomena of the underlying disease process, and further work is required to unearth the mechanisms of the differential clinical phenotypes.

## Introduction

Sjögren’s syndrome (SS) is a systemic autoimmune disease characterised by sicca symptoms, fatigue, autoantibodies, B cell hyper-reactivity and variable presentations of extra-glandular manifestations including neuropathy, cryoglobulinaemic vasculitis and lymphoma. Despite being described for many decades, no effective and specific treatments exist and management is focused on alleviating troubling symptoms and addressing organ-threatening complications (1). IgG autoantibodies against the Ro/La ribonucleoprotein nuclear complex are highly characteristic in SS and forms part of the diagnostic criteria for this disorder (2). These autoantibodies are frequented in other autoimmune disorders such as primary biliary cirrhosis and systemic lupus erythematosus (SLE) (3). Seroreactivity in SS is heterogeneous with the majority of patients displaying combined reactivity to Ro52 and Ro60 (58%), with or without anti-La antibodies. However, a smaller percentage of patients target either Ro52 or Ro60 alone (15% and 17% respectively) (4) and approximately 10% of cases are seronegative (4). Anti-La antibodies exist in around 38% of SS patients (5). Despite this serological variability, little is known about clinical subtyping patients based on anti-Ro/La status.

Ro52 is a cytoplasmic protein that functions as an Fc receptor and E3 ubiquitin ligase (6, 7). Anti-Ro52 antibodies (henceforth IgG isotype unless otherwise specified) have been associated with other autoimmune disorders, infection and malignancies (8, 9) and frequently associate with anti-Ro60 and/or anti-La autoantibodies. We and others have evaluated patients from a general laboratory cohort that tested positive for anti-Ro52 and found that patients with isolated anti-Ro52 (anti-Ro52 without anti-Ro60 and/or anti-La) had distinct laboratory and clinical features compared to those with combined anti-Ro52/Ro60/La reactivity (9, 10). Immunoassays in the twentieth century favoured detection of antibodies to SSA/Ro60, and therefore, missed the detection of anti-Ro52 autoantibodies which may not form immunoprecipitins (11). More modern assays with targeted anti-Ro52 detection (e.g., line immunoassays) now allow rapid and sensitive detection of this autoantibody.

In SS, anti-Ro52 positivity may signify a severe subset of patients associated with rheumatoid factor (RhF) positivity (12, 13). However, the value of stratifying SS patients by patterns of serum autoantibodies (anti-Ro52, anti-Ro60 and anti-La) has not been widely appreciated and inconsistencies remain in the literature. For example, one study found SS patients with isolated anti-Ro52 had higher degrees of sicca symptoms over other subsets (14), but this was not recapitulated in an earlier study (4). Given the putative links of anti-Ro52 with severe pathology and incongruities in the literature, we evaluated autoantibody subsets in SS patients. We hypothesise that patients with isolated anti-Ro52 represent a clinical subset of SS with molecularly distinct anti-Ro52 autoantibodies compared to patients with combined anti-Ro52/Ro60/La. To test this hypothesis, we evaluated the clinical features of anti-Ro52-positive SS patients with and without anti-Ro60/La in a single centre in Sydney, Australia. We also evaluated the molecular features of anti-Ro52 autoantibodies in different serological subsets by enzyme-linked immunosorbent assay (ELISA) and mass spectrometry (MS).

## Methods

### Patients

SS patients, diagnosed as per the American European Consensus Group (AECG) diagnostic criteria (2), were identified from the Department of Clinical Immunology & Allergy (Westmead Hospital) that attended between 2019 and 2022. The AECG 2002 criteria has excellent agreement with the more modern American College of Rheumatology-European League Against Rheumatism 2016 criteria (15, 16). Only primary SS patients (patients without other underlying autoimmunity) were considered. The census date was 1 April 2022.

Seroreactivity (anti-Ro52, anti-Ro60 and anti-La) were defined as per line immunoassay (LIA) (Euroimmun, Germany) at the Immunopathology Laboratory (Westmead Hospital). Healthy controls, that had no known comorbidities, were also recruited. Informed consent was obtained for collection of sera, which was stored at -80°C until ready for use. Laboratory and clinical data were extracted from medical records. Haematological parameters, SS clinical manifestations and disease activity were defined as per the European Alliance of Associations for Rheumatology (EULAR) SS disease activity index (ESSDAI) (17). ESSDAI measurements refer to the assessment at the patient’s last review.

### Enzyme-linked immunosorbent assay

An in-house indirect ELISA was developed using recombinant Ro52 (Arotec Diagnostics Ltd, New Zealand). Purity of Ro52 was determined to be >90% as determined by sodium dodecyl-sulfate polyacrylamide gel electrophoresis (SDS-PAGE). Antigens were coated on ELISA plates (Nunc MaxiSorp) at 1 µg/mL in PBS overnight. No-antigen controls were also run. Following blocking with 1% bovine serum albumin (BSA)/PBS for 1 hour at 37°C, the plate was washed x4 in 0.05% polysorbate 20/PBS. Sera were incubated for 30 min at 37°C, diluted 1:400 in 0.1% BSA/PBS and washed x5. Goat anti-human IgG, IgA or IgM conjugated to alkaline phosphatase (Sigma) were added to relevant wells, diluted 1:1000 in 1% skim milk/PBS and incubated for 30 min at 37°C. The plates were washed x4 and developed using 4-nitrophenyl phosphate (Sigma) 1 mg/mL in diethanolamine buffer. Plates were read at 15 min and 405 nm using a SpectraMax microplate reader, and OD values subtracted from specimen-specific no-antigen controls. The intra- and inter-assay coefficient of variation were 3.4% and 7.5% respectively. Non-linear regression analysis revealed good agreement with LIA densitometry with R^2^ = 0.81.

### Anti-Ro52 purification and mass spectrometric analysis

Serum anti-Ro52 were purified using Ro52 protein (Arotec Diagnostics, New Zealand)-coupled magnetic beads (Dynabeads MyOne Streptavidin T1, Invitrogen). Briefly, 25 µg of Ro52 were incubated with 50 µL magnetic beads (0.5 mg/mL) per 100 µL serum on a rotator for 30 min at room temperature. After coating, the beads were washed 3 times in 0.1% BSA/PBS and then incubated with diluted serum on a rotator for 2 h at room temperature. After incubation, the beads were washed 4 times with PBS and the bound antibodies were eluted with an elution buffer (100 mM glycine and 0.1% sodium deoxycholate, pH 11.5) for 5 min at room temperature. Purified antibodies were verified by running the protein on non-reduced Mini-Protean TGX stain-free SDS-PAGE gels (Bio-Rad). Anti-Ro52 IgG bands (150 kDa) were excised and subjected to trypsin and chymotrypsin (ThermoFisher Scientific) enzymatic digestion. Digested peptides were analysed with an Orbitrap Exploris 480 mass spectrometer (Thermo Scientific) coupled to an Ultimate 3000 UHPLC (Dionex, USA). Anti-Ro52 peptide sequences were analysed by *de novo* sequencing and International ImMunoGeneTics (IMGT) database matching using Peaks studio XPro software (Bioinformatics Solution Inc., Canada) as previously described (18, 19). Purification of anti-Ro52 autoantibodies from each individual serum was performed on at least two independent occasions, and the purified immunoglobulins digested by trypsin and chymotrypsin from each purification were subjected to mass spectrometry as two technical replicates, respectively.

### Statistics

Simple descriptive statistics were calculated for categorical variables. For continuous variables, Shapiro-Wilk test was used to test normality. Student’s t test or Mann-Whitney tests were used, as appropriate, to calculate differences in continuous variable means or medians between two groups. For multiple comparisons, the Kruskal-Wallis test with Dunn’s *post-hoc* test or Chi-squared tests were used. Anti-Ro52 antibody titres were compared to continuous variables by Spearman’s rho. SPSS version 22 statistical software was used for statistical calculations. A *p* value < 0.05 was considered significant.

### Ethics

Ethics approval was granted by the Western Sydney Local Health District Human Research Ethics Committee (2020/ETH01030).

## Results

### Autoantibody subsets and clinical features in Sjögren’s syndrome

To test the hypothesis that SS patients with isolated anti-Ro52 autoantibody profile had distinct clinical and laboratory features, we examined a cohort of SS patients at a single centre in Sydney, Australia. In our cohort, there were 123 SS patients; 114 (93%) were female and 113 (92%) were seropositive (contained autoantibodies to Ro52, Ro60 and/or La). Amongst the seropositive patients, 44 (36%) were anti-Ro52^+^Ro60^+^La^+^, 39 (32%) anti-Ro52^+^Ro60^+^La^−^, 15 (12%) anti-Ro52^+^Ro60^−^ La^−^, 13 (11%) anti-Ro52^−^Ro60^+^La^−^, 1 (1%) anti-Ro52^+^Ro60^−^La^+^ and 1 (1%) anti-Ro52^−^Ro60^+^La^+^.

The median age of the patients at last review was 57 years (range 20 – 88), and a median age of symptom onset of 43 years (13 – 80). We stratified patients into isolated anti-Ro52 (anti-Ro52^+^Ro60^−^ La^−^), seronegative (anti-Ro52^−^Ro60^−^La^−^), isolated anti-Ro60, and various combinations of combined anti-Ro52 subsets (anti-Ro52^+^Ro60^+^La^+^, anti-Ro52^+^Ro60^+^La^−^ and anti-Ro52^+^Ro60^−^La^+^) SS subsets (**Table 1**). No other differences were seen in sex distribution and age apart from isolated anti-Ro52 patients having a later onset of symptoms and possibly being slightly older (**Table 1**). More patients in the isolated anti-Ro52 group were on hydroxychloroquine and prednisolone treatment than patients in the other groups (**Table 1**). Patients in the isolated anti-Ro52 subset tended to present later but are followed up earlier than their SS counterparts (**Table 1**).

**Table 1:** Demographic and treatment characteristics of Sjögren’s syndrome patients.

	Isolated anti-Ro52 (*n* = 15)	Seronegative (*n* = 10)	Non-isolated anti-Ro52				
			Isolated anti-Ro60 (*n* = 13)	Anti-Ro52 + anti-Ro60 (*n* = 39)	Anti-Ro52 + anti-Ro60 + anti-La (*n* = 44)	All non-isolated anti-Ro52 (*n* = 108)	Comparison between non-isolated anti-Ro52 subsets† (*p* value)
Female (n) (%)	13 (87)	10 (100)	13 (100)	36 (92)	40 (91)	101 (94)	0.220
Age at last review (years) (mean ± SD)	62.2 ± 17.4	58.7 ± 17.8	52.5 ± 16.2	51.3 ± 14.4*	57.5 ± 16.8	54.6 ± 15.9	0.202
Age at symptoms (years) (mean ± SD)	53.5 ± 17.5	45.3 ± 20.0	40.3 ± 16.6	40.1 ± 12.4*	45.8 ± 15.6	42.9 ± 14.8*	0.380
Duration of disease from symptoms to diagnosis (years) (mean ± SD)	6.8 ± 4.1	11.5 ± 10.5	9.2 ± 6.0	10.9 ± 9.5	10.6 ± 8.5	10.7 ± 8.6	0.996
Duration of disease from symptoms to first review (years) (mean ± SD)	5.4 ± 4.3	10.5 ± 11.2	2.1 ± 2.2*	4.3 ± 7.1	2.4 ± 3.4**	3.1 ± 5.1**	0.532
Duration from diagnosis to last review (years) (mean ± SD)	2.2 ± 2.5	1.7 ± 1.4	8.8 ± 7.5**	8.2 ± 9.5*	8.7 ± 8.5**	8.5 ± 8.6**	0.824
Treatments							
Hydroxychloroquine Prednisolone Mycophenolate Rituximab Methotrexate Sulfasalazine Azathioprine	15 (100) 5 (33) 1 (7) 1 (7) 0 (0) 0 (0) 1 (7)	3 (30)*** 2 (20) 0 (0) 0 (0) 0 (0) 0 (0) 0 (0)	3 (23) 2 (15) 2 (15) 2 (15) 2 (15) 2 (15) 0 (0)	13 (33)* 4 (10) 3 (8) 1 (3) 3 (8) 0 (0) 1 (3)	13 (30)* 3 (7)* 2 (5) 1 (2) 2 (5) 0 (0) 0 (0)	33 (31)**** 11 (10)* 7 (7) 4 (4) 7 (7) 2 (2) 1 (1)	0.662 0.278 0.841 0.508 0.841 0.102 0.408

Column statistics (denoted by asterisks) show statistically significant differences for each subset when compared to the isolated anti-Ro52 subset (Mann-Whitney). Differences between non-isolated anti-Ro52 subsets were calculated Kruskal-Wallis test with Dunn’s post-hoc test. SD, standard deviation. *p < 0.05. **p < 0.01. ***p < 0.001. ****p < 0.0001.

† These include seronegative, isolated anti-Ro60, anti-Ro52 + anti-Ro60, and anti-Ro52 + anti-Ro60 + anti-La four subsets.

Next, we reviewed the laboratory and clinical differences between the isolated anti-Ro52 and other serological subtypes. We analysed the seronegative, isolated anti-Ro60, anti-Ro52/Ro60, anti-Ro52/Ro60/La and all non-isolated anti-Ro52 combined subsets (**Table 2**). Interestingly, amongst seropositive SS patients, the isolated anti-Ro52 subset identified SS patients associated with cryoglobulinaemia, RhF, low C4 complement, peripheral nervous system involvement, Raynaud’s phenomena, cutaneous vasculitis, lymphadenopathy and pulmonary involvement. Five of the 7 cases of cryoglobulinaemia were type II cryoglobulins (4 monoclonal IgMκ, and 1 monoclonal IgAκ RhF); the remaining 2 patients’ cryoglobulins were too small (cryocrit ≤ 1%) to be accurately typed. The isolated anti-Ro52 and anti-Ro60-only patients had a lower incidence of hypergammaglobulinaemia (defined as IgG > 16.0 g/L) than all other seroreactive groups. Consequently, isolated anti-Ro52 SS patients overall had a higher ESSDAI compared to other subsets (**Table 2**) which may explain the higher use of systemic immunosuppressants in these patients (**Table 1**). On comparing ESSDAI with the age of first symptoms, duration of disease from symptoms to first review, and duration of disease to formal diagnosis, there was no significant association by Spearman’s ρ (*p >* 0.05), indicating no relationship between disease activity and the length of patients’ disease. Further analyses comparing the four non-isolated anti-Ro52 subsets (seronegative, anti-Ro60 only, anti-Ro52/Ro60 and anti-Ro52/Ro60/La) were also performed showing largely clinically and laboratorily homogeneous populations except for a greater degree of rheumatoid factor positivity and hypergammaglobulinaemia in the anti-Ro52/Ro60/La subset (**Table 2**).

**Table 2:** Laboratory and clinical parameters in seropositive Sjögren’s syndrome patients, stratified by anti-Ro52 status.

	Isolated anti- Ro52 (*n* = 15)	Seronegative (*n* = 10)	Non-isolated anti-Ro52				
			Isolated anti- Ro60 (*n* = 13)	Anti-Ro52 + anti- Ro60 (*n* = 39)	Anti-Ro52 + anti-Ro60 + anti-La (*n* = 44)	All non-isolated anti-Ro52 SS patients (*n* = 108)	Comparison between non- isolated anti-Ro52 subsets† (*p* value)
Immunological parameters							
Anti-Ro52 titre (units) (mean ± SD) Positive rheumatoid factor Rheumatoid factor titre (IU/mL) (mean ± SD) Low C4 complement Hypergammaglobulinaemia Cryoglobulinaemia Monoclonal gammopathy ESR (mm/hr) (mean ± SD)	83.7 ± 43.1 7/15 (47) 130.7 ± 178.9 6/12 (50) 2/15 (13) 7/13 (54) 5/15 (33) 20.1 ± 16.2	– 1/8 (13)^a^ 10.2 ± 6.7^a^ 0/6 (0)* ^a^ 0/2 (0)^a^ 0/7 (0) 0/5 (0) 26.1 ± 22.1^a^	– 1/13 (8)* ^a^ 7.3 ± 4.9* ^a,b^ 0/11 (0)* ^a^ 2/10 (20)^a,b^ 0/6 (0)* 0/9 (0) 17.4 ± 21.2^a,b^	82.1 ± 30.7^a^ 15/37 (41)^b^ 40.1 ± 58.5** ^a,c^ 3/37 (8)** ^a,b^ 17/36 (47)* ^a,b^ 1/18 (6)** 4/34 (12) 18.0 ± 16.5^a,b^	100.6 ± 16.4^b^ 25/43 (58)^b^ 46.8±53.1** ^a,c^ 11/42 (26)^a,c^ 29/39 (74)**** ^a,c^ 0/26 (0)**** 7/41 (17) 35.3 ± 26.3* ^a,c^	79.5 ± 38.8 42/103 (41) 38.0 ± 52.6*** 14/101 (14)** 49/94 (52)** 1/56 (2)**** 11/96 (12) 25.8 ± 23.3	0.003 0.003 < 0.001 0.024 < 0.001 0.553 0.300 0.001
Laboratory parameters							
Anaemia (haemoglobin < 120 g/L) Thrombocytopaenia (platelets < 150 x 10^9^/L) Neutropaenia (neutrophils < 1.5 x 10^9^/L) Lymphopaenia (lymphocytes < 1.0 x 10^9^/L) Estimated glomerular filtration rate (mL/min/1.73m^2^ mean ± SD)	4/15 (27) 1/15 (7) 2/15 (13) 6/15 (40) 79.4 ± 19.8	2/10 (20) 0/10 (0) 0/10 (0) 0/10 (0) 72.0 ± 21.0	1/13 (8) 0/13 (0) 0/13 (0) 1/13 (8) 88.2 ± 5.8	5/39 (13) 1/39 (3) 3/39 (8) 6/39 (15) 82.1 ± 14.2	12/44 (27) 4/44 (9) 8/44 (18) 8/44 (18) 77.9 ± 20.0	19/108 (18) 6/108 (6) 12/108 (11) 16/108 (15)* 80.3 ± 17.1	0.199 0.332 0.119 0.435 0.099
Clinical parameters							
ESSDAI (mean ± SD) Keratoconjunctivitis sicca (patient reported) Average Schirmer’s test (mm) (mean ± SD) Xerostomia Salivary flow rate (mL/min) (mean ± SD) Central nervous system involvement Peripheral nervous system involvement Raynaud’s phenomena Cutaneous vasculitis Digital purpura Arthralgias/Arthritis Myositis Lymphadenopathy Lymphoma MALT lymphoma Other marginal zone lymphoma Diffuse large B cell lymphoma Burkitt lymphoma T cell lymphoma Pulmonary involvement Renal involvement Constitutional symptoms Fatigue (patient-reported in last 2 weeks)	15.9 ± 13.5 13/15 (87) 9.2 ± 11.9 14/15 (93) 0.15 ± 0.22 0/15 (0) 3/15 (20) 7/14 (50) 7/14 (50) 2/14 (14) 9/14 (64) 0/14 (0) 5/14 (36) 3/15 (20) 2/15 (13) 1/15 (7) 0/15 (0) 0/15 (0) 0/15 (0) 3/14 (21) 3/15 (33) 2/14 (1) 10/14 (71)	3.3 ± 4.2** 10/10 (100) 6.2 ± 3.9 9/10 (90) 0.12 ± 0.10 2/10 (20) 0/10 (0) 0/10 (0)* 0/10 (0)* 0/10 (0) 1/10 (10)* ^a^ 1/10 (0) 0/10 (0) 0/10 (0) 0/10 (0) 0/10 (0) 0/10 (0) 0/10 (0) 0/10 (0) 1/10 (0) 0/10 (0) 0/10 (0) 5/8 (63)	6.9 ± 8.0* 12/12 (100) 11.9 ± 12.1 12/12 (100) 0.20 ± 0.0 2/13 (15) 2/13 (15) 1/11 (9)* 0/39 (0)**** 1/13 (8) 7/13 (54)^a,c^ 1/13 (8) 1/13 (8) 0/13 (0) 0/13 (0) 0/13 (0) 0/13 (0) 0/13 (0) 0/13 (0) 1/13 (8) 0/13 (0) 2/13 (15) 8/9 (89)	5.9 ± 5.4* 35/39 (90) 13.6 ± 9.7 34/39 (87) 0.17 ± 0.15 6/39 (15) 4/39 (10) 5/36 (14)* 0/13 (0)** 1/39 (3) 23/39 (59)^b,c,d^ 0/39 (0) 4/39 (10)* 3/37 (8) 1/37 (3) 0/37 (0) 1/37 (3) 0/37 (0) 1/37 (3) 2/39 (5) 1/39 (3) 3/39 (8) 23/28 (82)	5.5 ± 5.0* 40/44 (91) 11.2 ± 8.3 42/44 (96) 0.15 ± 0.14 3/44 (7) 1/44 (2)* 7/43 (16)* 2/43 (5)*** 6/43 (14) 16/44 (36)^a,c,e^ 0/44 (0) 1/44 (2)** 2/43 (5) 0/43 (0) 1/43 (2) 0/43 (0) 1/43 (2) 0/37 (0) 0/44 (0)* 4/44 (9) 2/44 (5) 29/39 (74)	5.6 ± 5.5** 99/107 (93) 10.6 ± 8.9 99/107 (93) 0.15 ± 0.13 13/108 (12) 7/108 (7) 14/102 (14)** 2/107 (2)**** 8/107 (8) 48/108 (44) 2/108 (2) 6/108 (6)** 6/105 (6) 1/105 (1) 1/105 (1) 2/105 (2) 1/105 (1) 1/105 (1) 4/108 (4)* 5/108 (5) 7/108 (7) 66/86 (77)	0.383 0.506 0.054 0.368 0.836 0.524 0.211 0.557 0.401 0.196 0.021 0.057 0.363 0.567 1.000 0.400 1.000 0.400 1.000 0.316 0.332 0.439 0.520

Data are presented as proportions (%) unless otherwise specified. Statistics are compared to the isolated anti-Ro52 subset (reference subset). Variables with different superscripted lowercase letters are significantly different from each other (p < 0.05). SD, standard deviation. ESR, erythrocyte sedimentation rate. ESSDAI, EULAR Sjögren’s syndrome disease activity index. MALT, mucosa-associated lymphoid tissue. *p < 0.05, **p < 0.01, ***p < 0.001, ****p < 0.0001.

† These include seronegative, isolated anti-Ro60, anti-Ro52 + anti-Ro60, and anti-Ro52 + anti-Ro60 + anti-La four subsets.

**Figure 1:**
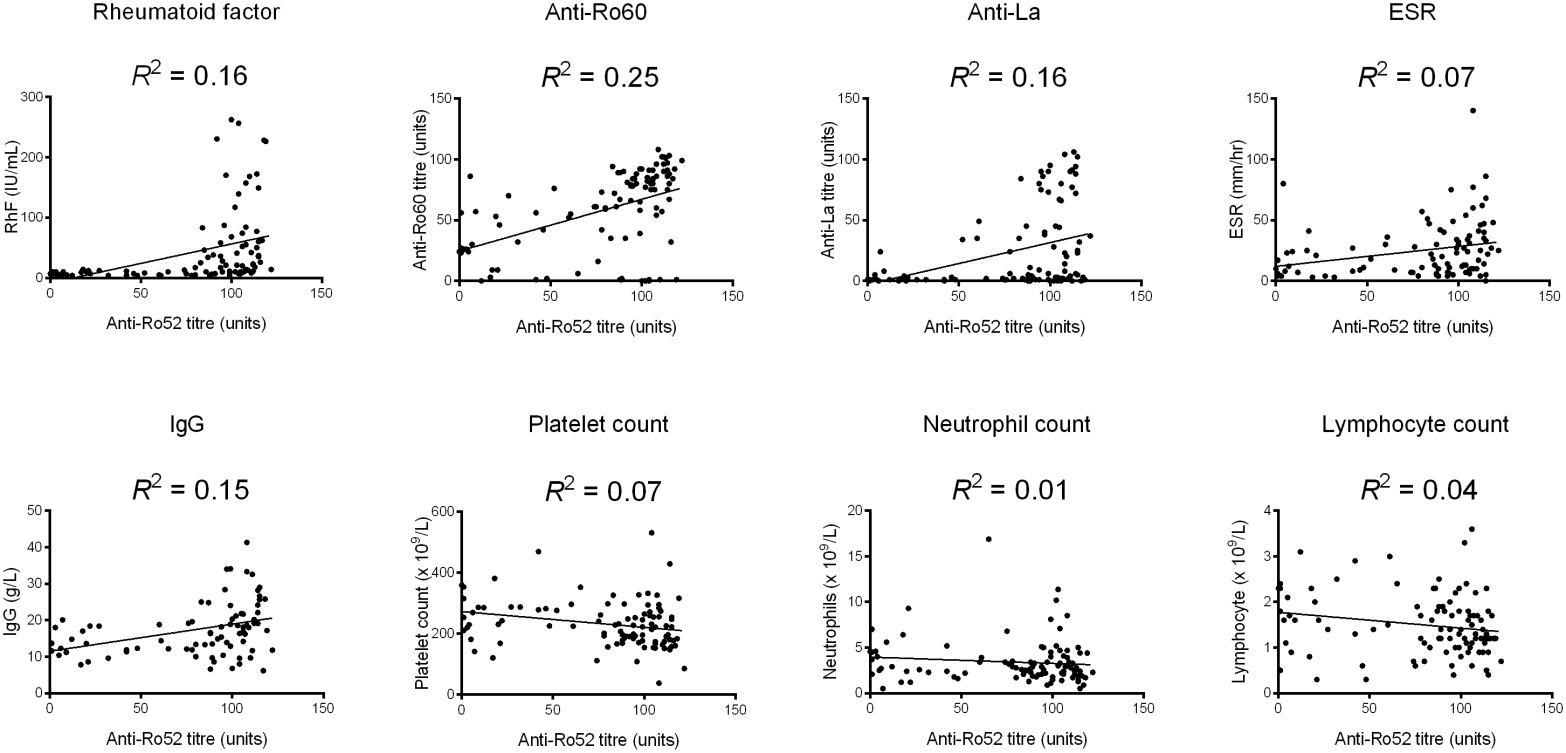
Scatterplots of significant correlations between anti-Ro52 and continuous variables. A linear regression line has been added as well as a goodness-of-fit statistic (R^2^). *ESR*, erythrocyte sedimentation rate.

For continuous variables, anti-Ro52 titre was correlated with anti-Ro60 titre (Spearman’s ρ = 0.498, *p* < 0.001), anti-La titre (ρ = 0.386, *p* < 0.001), RhF titre (ρ = 0.604, *p* < 0.001), erythrocyte sedimentation rate (ESR) (ρ = 0.351, *p* < 0.001), IgG level (ρ = 0.379, *p* < 0.001), platelet count (ρ = -0.305, *p* = 0.002), neutrophil count (ρ = -0.208, *p* = 0.035) and lymphocyte count (ρ = -0.225, *p* = 0.022). Scatterplots of these variables along with regression lines are shown in **Figure 1**. There were no significant correlations (*p* > 0.05) for ESSDAI, haemoglobin, estimated glomerular filtration rate (eGFR), unstimulated salivary flow rate nor average Schirmer’s test measurement (data not shown).

### Anti-Ro52 serology in Sjögren’s syndrome

**Figure 2:**
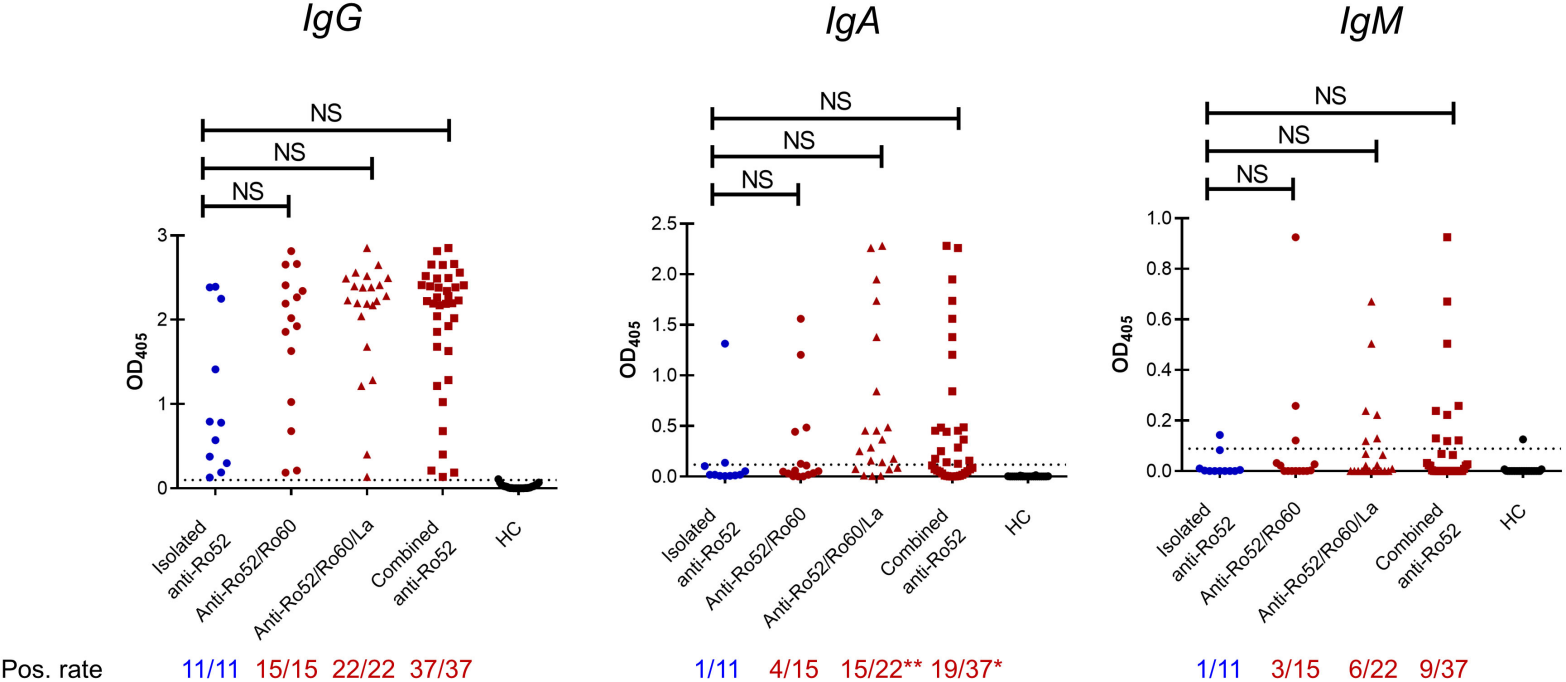
Anti-Ro52 serological subsets in Sjögren’s syndrome patients. Forty-eight patients’ sera were analysed for anti-Ro52 IgG, IgA and IgM *via* enzyme-linked immunosorbent assay (ELISA). Twenty-five healthy controls (HC) were used to establish the cut-off optical density (OD), defined as mean OD + 2 standard deviations. Dotted lines on the *y* axis represent the cut-off OD. All statistics are compared to the isolated anti-Ro52 subset (blue), using Kruskal-Wallis and Dunn’s *post-hoc* test, and Fisher’s exact test for continuous and non-continuous variables respectively. *NS*, not significant by Kruskal-Wallis tests.

Given that patients with isolated anti-Ro52 had distinct laboratory and clinical findings compared to other serological subsets, we next investigated whether their anti-Ro52 autoantibodies exhibited differences in immunoglobulin class switching. Forty-eight SS patients’ sera were available for evaluation and were stratified according to the presence or not of anti-Ro60 and anti-La with anti-Ro52 (**Figure 2**). All patients contained IgG autoantibodies reactive with full length recombinant Ro52; those with isolated anti-Ro52 autoantibodies and anti-Ro60 showed less switching to IgA than those with anti-La (**Figure 2**). Median anti-Ro52 titres in patients specifically positive for IgA and IgM anti-Ro52 were compared across the subsets revealing no significant differences (data not shown). Overall, there were no differences for anti-Ro52 antibody titres between all subsets by ELISA (IgA, IgM and IgG isotypes) or LIA densitometry (IgG isotype) (**Table 2**).

### Proteomic analysis of anti-Ro52

**Figure 3:**
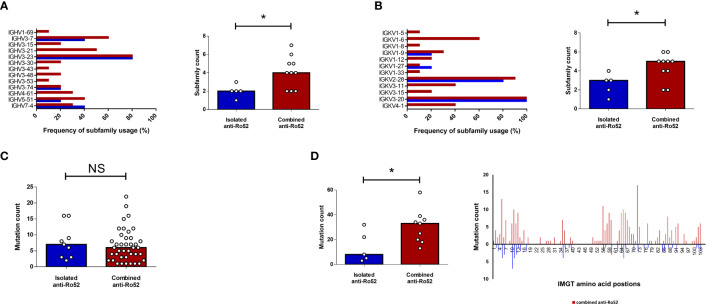
Anti-Ro52 IgG proteomic analyses by mass spectrometry. Five isolated (blue) and ten combined (red) anti-Ro52 Sjögren’s syndrome patients’ anti-Ro52 were analysed according to their heavy (IGHV) **(A)** and light (IGKV) **(B)** chain subfamily composition. Frequency of subfamily usage refers to the percentage of patients using the designated subfamily within the isolated or combined anti-Ro52 subsets. Column graphs represent median values. **(C)** Absolute frequency (count) of amino acid mutations in each IGHV subfamily were quantified for each subset. Each circle represents one subfamily. **(D)** Repeat frequency analyses were then restricted to the heavy complementarity-determining regions (HCDR) 2 and 3 combined. *p < 0.05. *NS*, not significant. *IMGT*, international ImMunoGeneTics information database. Mann-Whitney U test was used for analyses.

The identification of distinct serological and clinical subsets in SS raises the question of whether anti-Ro52 autoantibodies are molecularly different in patients with isolated versus combined anti-Ro52. To compare anti-Ro52 autoantibody molecular characteristics, including immunoglobulin variable region usage and somatic hypermutation, in patients with isolated and combined serological profiles, we subjected 15 SS patients’ anti-Ro52 IgG to mass spectrometric (MS) analyses. We compared 5 isolated anti-Ro52 SS patients to 10 combined anti-Ro52 (comprising 2 anti-Ro52/Ro60 and 8 anti-Ro52/Ro60/La SS patients). All anti-Ro52 were IgG1-kappa isotype by MS. The isolated anti-Ro52 subset displayed more restricted heavy (IGHV) and light (IGKV) chain subfamily diversity compared to the combined anti-Ro52 group when comparing the number of unique IGHV and IGKV regions per patient. Individuals with isolated anti-Ro52 used between 1 and 4 unique IGHV and IGKV gene segments compared to the combined anti-Ro52 with 2 to 7 different IGHV and IGKV regions (**Figures 3A, B** respectively). The overall number of amino acid substituting mutations was comparable between isolated and combined anti-Ro52 subsets with both groups exhibiting a variable range of mostly germline encoded anti-Ro52 to mutated (**Figure 3C**). However, when specifically evaluating the heavy chain complementarity-determining region (HCDR) 2 and 3 regions, we found a greater degree of amino acid substitutions in the combined anti-Ro52 subset (**Figure 3D**). We did not observe any relationship between ESSDAI and the frequency of heavy chain (ρ = -0.123, *p* = 0.700) and light chain (ρ = -0.522, *p* = 0.200) subfamily usage; and ESSDAI and average heavy chain mutation frequency (ρ = 0.200, *p* = 0.783).

## Discussion

Autoantibodies targeting Ro52, Ro60 and La are characteristic of SS; however, it is unclear whether different serological profiles associate with specific manifestations in this clinically diverse disease. Herein, we describe a subset of patients with SS who were positive for anti-Ro52 without anti-Ro60/La (isolated anti-Ro52) with higher disease activity (ESSDAI) and increased incidence of cryoglobulinaemia, compared to patients with anti-Ro60/La and those that were seronegative to these autoantibodies. These findings have important prognostic implications and highlight the importance of accurate serological phenotyping and making the distinction between autoantibodies to Ro52 and Ro60, which are often co-reported as “anti-SSA/Ro” (3).

The absence of these autoantibodies appears to have clinical relevance as well. Some studies point to seronegative patients displaying a milder disease phenotype compared to seropositive SS patients; yet, may still have significant sicca, pain and fatigue symptoms (20, 21). Our seronegative cohort appeared to have lower ESSDAI and objective markers of sicca (**Table 2**); however, this did not reach statistical significance perhaps due to the relatively few number of patients in our seronegative cohort.

It is interesting to note the differences in ages and durations of disease in our subsets (**Table 1**). Despite the similar ages at last review across the subsets in this cohort, the isolated anti-Ro52 subset developed symptoms later than the rest of the cohort. This is consistent with the finding that SS patients with anti-Ro60 and anti-La tend to develop symptoms earlier (22, 23). Curiously, in our cohort, we did not find any association between age of presentation and disease duration, and ESSDAI, while another study reported that early-onset SS presents more severely (22). This discrepancy may be explained by the cross-sectional nature of our study; we only recorded the ESSDAI at last review rather than longitudinally analyse disease activity for each patient. Finally, it is also noteworthy that the isolated anti-Ro52 SS patients tend to present slightly later for clinical review following the onset of symptoms. It is not clear from our study why this may be as one would presume that these patients would present earlier for review given the severity of their disease. However, in one cohort, older patients with symptoms of SS experience a longer delay for a formal diagnosis compared to their younger counterparts, which would be in keeping with our findings (24).

Identification of anti-Ro52 has been shown to have prognostic implications in other diseases. For example, in scleroderma (systemic sclerosis), anti-Ro52 is an independent predictor of all-cause mortality, and identifies individuals at a greater risk of pulmonary involvement (25, 26). Patients with isolated anti-Ro52 (no other scleroderma autoantibodies) portend worse prognoses (26). In contrast, isolated anti-Ro52 in patients with undifferentiated connective tissue disease identified a milder phenotype consisting primarily of articular and haematological manifestations (27). Although it may be tempting to speculate the role for the autoantibody in modulating immunological processes, in reality, it is likely an immunological epiphenomenon that reflects the underlying immunopathogenesis.

Anti-Ro52 autoantibodies in the absence of anti-Ro60/La appear to be distinct from anti-Ro52 combined with anti-Ro60/La by exhibiting reduced isotypes, immunoglobulin variable region diversity and amino acid mutations. What may account for the serological and molecular differences of the anti-Ro52 subsets? The human leukocyte antigen (HLA) class II locus (e.g., HLA-DR3) has been strongly implicated in intermolecular epitope spreading to Ro and La autoantigens (28). SS patients with anti-Ro52 and anti-Ro60/La may have a specific HLA-II haplotype that allows more effective antigen presentation to T and B cells; but whether this also influences isotype switching and/or molecular diversification is unknown. It is also possible that different forms of Ro52 antigen are presented and driving distinct autoantibody responses, similar to what was proposed for different anti-Ro60 responses observed in SS and SLE (29–31).

In a Belgian cohort, Deroo et al. (14) found the main distinguishing feature of their isolated anti-Ro52 cohort to be more pronounced subjective and objective markers of sicca symptoms. In contrast, a cohort from the United States failed to find any significant differences in sicca symptoms (4), in line with our results (**Table 2**). Interestingly, isolated anti-Ro52 did not confer higher disease activity (ESSDAI) in another cohort of SS patients stratified by their serologies (4). Our markedly higher ESSDAI in our cohort may be explained by the manifestations of cryoglobulinaemia including cutaneous vasculitis and Raynaud’s phenomena (**Table 2**). The incidence of cryoglobulinaemia was not explored in these earlier studies (4, 14); however, the isolated anti-Ro52 subset positively correlated with RhF (4, 12, 14), consistent with our findings (**Table 2**). Moreover, a cross-sectional study in a laboratory cohort of SS patients that measured cryoglobulins in serological subsets, found that isolated anti-Ro52 antibodies were associated with cryoglobulinaemia (32).

The unusually high frequency of cryoglobulinaemia with isolated anti-Ro52 in this study is intriguing with 54% having type II mixed cryoglobulinaemia compared to 1-6% in other serological subsets (**Table 2**). In another cohort of SS, the incidence of cryoglobulinaemia was around 10% (33). Cryoprecipitating RhFs have been shown to evolve from soluble RhF through the accumulation of somatic hypermutations that increase the propensity to form insoluble aggregates (34). Hence the positive correlation between anti-Ro52 and RhF autoantibodies may be relevant to the increased incidence of cryoglobulinaemia in this group. Why the absence of anti-Ro60/La may increase the risk of cryoglobulinaemia is mechanistically not clear. Previous studies have proposed anti-Ro/La autoantibodies complexed with Ro/La ribonucleoproteins may activate RhF B cells by simultaneously engaging the B cell receptor and Toll-like receptors (35–37). The clinical findings presented herein may suggest that anti-Ro52 antibodies are a more potent stimulator of RhF B cells than anti-Ro60 and anti-La autoantibodies. Alternatively, there may be specific molecular properties of anti-Ro52 autoantibodies that increase the propensity of secreted RhF immune complexes to precipitate. Further studies are required to unravel these mechanisms.

In cohort studies, rituximab is highly effective and generally safe in the treatment against non-viral cryoglobulinaemic vasculitis (38). It is therefore surprising that despite the relatively high prevalence of cryoglobulinaemia in the isolated anti-Ro52 cohort, that only 1 patient (7%) was on rituximab (**Table 1**). This discrepancy may be explained by two patients with isolated anti-Ro52 having asymptomatic cryoglobulinaemia and therefore not needing treatment. Moreover, funding (Medicare) restrictions for rituximab in Australia do not readily support use of this agent as sole first-line therapy for non-viral cryoglobulinaemic vasculitis. Since the census of the study (April 2022), three additional SS with isolated anti-Ro52 and cryoglobulinaemic vasculitis have successfully received rituximab after failing first-line therapy.

This study has several limitations. Firstly, as a cross-sectional study, the ability to understand the anti-Ro52 response longitudinally is limited. It is important to confirm the serological stability of the subsets and identify whether the molecular features of anti-Ro52 autoantibodies change over time. In addition, this is a single-centre study performed in one part of Sydney, Australia which has distinct ethnic and sociodemographic characteristics from other parts of Sydney and Australia. Whether the clinical characteristics of the serological subsets are upheld with other SS cohorts would need to be investigated in future studies. Furthermore, the relatively small isolated anti-Ro52 affects the generalisability of our results to other centres and SS cohorts; as such, these findings should be examined in additional cohorts, preferably with contrasting ethnic characteristics. Finally, whilst the association between anti-Ro52 and cryoglobulinaemia is clinically important, our current study is unable to offer mechanistic insight into the pathogenesis of this association. Elucidating whether Ro52/TRIM21 and anti-Ro52 have a specific role in the pathogenesis of cryoglobulinaemia should be the focus for future research. Indeed, understanding the origins of pathogenic autoantibodies and failed immunological tolerance would be instrumental in developing targeted therapies for SS patients.

## Data availability statement

The datasets presented in this study can be found in online repositories. The names of the repository/repositories and accession number(s) can be found below: PXD038765 and 10.6019/PXD038765 (ProteomeXchange).

## Ethics statement

The studies involving human participants were reviewed and approved by Western Sydney Local Health District Human Research Ethics Committee. The patients/participants provided their written informed consent to participate in this study.

## Author contributions

AL, TG, JJW and JR contributed to conception and design of the study. AL, JJW, TP, M-WL, SS, DS, TC and RT contributed to data collection and analyses. AL wrote the first draft of the manuscript. JR provided study supervision. All authors contributed to the article and approved the submitted version.
